# Identification of Genomic Instability in Cows Infected with BVD Virus

**DOI:** 10.3390/ani13243800

**Published:** 2023-12-09

**Authors:** Katarzyna Kępka, Ewa Wójcik, Anna Wysokińska

**Affiliations:** Institute of Animal Science and Fisheries, University of Siedlce, Prusa 14, 08-110 Siedlce, Poland; katarzyna.kepka@uph.edu.pl (K.K.); anna.wysokinska@uph.edu.pl (A.W.)

**Keywords:** dairy cows, Holstein-Friesian, BVD, genome instability, chromosome instability, reproductive disorders

## Abstract

**Simple Summary:**

The aim of the study was to identify the genomic instability in cows with reproductive disorders following infection with the BVD virus. The genomic stability was analyzed using the sister chromatid exchange, fragile sites, and comet assays. Statistically significant differences were noted between the groups. Of the three assays, the comet assay proved to be the most sensitive for identifying DNA damage in the animals.

**Abstract:**

An important factor for dairy cattle farmers is the profitability of cattle rearing, which is influenced by the animals’ health and reproductive parameters, as well as their genomic stability and integrity. Bovine viral diarrhea (BVD) negatively affects the health of dairy cattle and causes reproductive problems. The aim of the study was to identify genomic instability in cows with reproductive disorders following infection with the BVD virus. The material for analysis was peripheral blood from Holstein-Friesian cows with reproductive problems, which had tested positive for BVD, and from healthy cows with no reproductive problems, which had tested negative for BVD. Three cytogenetic tests were used: the sister chromatid exchange assay, fragile sites assay, and comet assay. Statistically significant differences were noted between the groups and between the individual cows in the average frequency of damage. The assays were good biomarkers of genomic stability and enabled the identification of individuals with an increased frequency of damage to genetic material that posed a negative impact on their health. The assays can be used to prevent disease during its course and evaluate the genetic resistance of animals. This is especially important for the breeder, both for economic and breeding reasons. Of the three assays, the comet assay proved to be the most sensitive for identifying DNA damage in the animals.

## 1. Introduction

An important factor for dairy cattle farmers is the profitability of cattle rearing, which is dependent on many factors. These include the animal’s health, reproductive parameters, age, and genetic determinants, but also herd management practices, such as feeding plans, means of detecting estrus, and the animals’ living environment [1,2,3,4,5,6]. Any deviations from these standards can negatively affect milk production [7,8,9]. Dairy cattle breeding has long been aimed at a high milk yield, often at the expense of functional traits, the loss of which may entail health disorders and affect longevity [10,11,12,13,14,15]. Animals with high genetic resistance maintain a good production level despite the frequently negative impact of exogenous factors [16,17]. Breeding programs currently emphasize improving the resistance, functional traits, and reproductive traits [18,19]. According to Ayane et al. [20] and Arero [6], a successful reproductive process includes well-functioning ovaries, normal estrous behavior, mating, the ability to conceive and nourish the embryo, the birth of viable young, the resumption of the estrous cycle, and the restoration of uterine function after parturition.

Some diseases can negatively affect reproduction in dairy cattle. One of these is bovine viral diarrhea (BVD). The virus inducing this disease belongs to the genus *Pestivirus* of the family *Flaviviridae*. It results in embryo resorption, abortion, the birth of weak calves, problems rearing them, low weight gain, increased treatment costs, prolonged calving intervals, and reduced milk yields [21,22]. The subclinical form of BVD is more common than the clinical form and lasts from 2–3 days to 4 weeks [23]. The symptoms of the clinical form depend on the strain of the virus and the animal’s immunity [22]. In cows that are not pregnant, reduced productivity, fever, diarrhea, hemorrhagic changes, pneumonia, and even fatal mucosal disease have been observed [24,25,26]. In pregnant cows, this virus can have teratogenic effects and can adversely affect embryo development and the maintenance and course of pregnancy [27,28,29]. Infection with this virus in early pregnancy (40–120 days) can lead to persistent infection of the calves, which can later result in culling to eliminate the BVD virus from the herd [8,30,31].

Intensified dairy production has led to a decrease in the genetic resistance of cattle [14,32,33,34]. Any disturbances of the genome stability and integrity caused by pathogens or mutagens adversely affect the functioning of the body [17,35]. Bovine viral diarrhea (BVD) has a cytopathogenic and lymphocytopathogenic course and is incurable. Protection against BVD is provided by vaccines. They are used prophylactically or when the virus is active in the herd to prevent the onset of symptoms of the acute form of the disease, reduce its severity, enrich colostrum with additional antibodies, reduce the number of intrauterine infections in cows and heifers, and prevent the birth of persistently infected animals. The vaccines should be given cyclically. They reduce the risk of abortion and fetal infection. This form of biosecurity is very important due to the high prevalence of the disease, which causes enormous financial losses [36].

Genomic stability can be tested using cytogenetic assays, which are highly sensitive biomarkers. They make it possible to identify damage to genetic material in an animal at various stages: pre-clinical, clinical during treatment, or after treatment. These tests include the comet assay, sister chromatid exchange assay (SCE), and fragile sites assay (FS), which can be used to monitor the level of generated damage. The comet assay (or single cell gel electrophoresis—SCGE) is a technique that can be used to assess the DNA integrity of any tissue, including lymphocytes [37]. In the cells subjected to electrophoresis, fragmented DNA migrates faster through the agarose matrix than intact DNA, creating an image reminiscent of the tail of a comet [38]. SCGE detects various types of DNA lesions, such as single-stranded and double-stranded DNA breaks, sites that are unstable in an alkaline environment, modified bases, incomplete repair sites, and cross-links between the strands [39].

The other two assays require in vitro cell cultures. The sister chromatid exchange assay identifies single- and double-strand DNA breaks caused by genotoxic and mutagenic factors interfering with replication [37,40]. They also have a destructive effect on the functioning of the checkpoints and DNA repair mechanisms. Unrepaired or incorrectly repaired DNA strand breaks can lead to destabilization and the rearrangement of genetic information, disturbing the integrity and stability of the genome and even leading to apoptosis [41,42,43,44]. Sister chromatid exchanges occur when DNA damage is not correctly repaired in the S phase of the cell cycle, which leads to an exchange of DNA duplexes in the sister chromatids of the dividing chromosome [45,46]. Fragile sites are also the result of erroneous replication and the mechanisms repairing disturbances in the progression of replication forks [47]. The ineffectiveness of these cellular mechanisms can result in gaps, breaks, or constrictions on the metaphase chromosomes under replication stress [48,49]. They are transferred between the generations of dividing cells, causing genomic instability [49]. They can be rare (RFS) or common (CFS) in the genome. The chromosome fragility at these sites is associated with the expansion of dinucleotide or trinucleotide repeats, which can form secondary DNA structures, affecting DNA replication and transcription [50,51,52,53]. They can also be a normal component of the chromosome structure, encompassing large genomic regions—even thousands of kilobases [54,55,56,57]. Early replicating fragile sites (ERFSs) are sensitive sites which colocalize with clusters of genes with a high expression and are enriched with repeated sequences. The fragility of ERFSs increases in the case of replication stress and ATR inhibition. They replicate early, are rich in GC pairs, and have a tendency to form secondary DNA structures, in which DNA is in the form of ssDNA. This arrests DNA replication and causes a replication–transcription conflict [50,58,59].

The aim of the study was to identify the genomic instability in Holstein-Friesian cows with reproductive disorders following infection with the BVD virus.

## 2. Materials and Methods

### 2.1. Animals

The research material was peripheral blood drawn from the tail vein of 40 female Holstein-Friesian cows. The age of the cows ranged from 2.5 to 5 years. Group 1 (G1) comprised cows that had tested positive for BVD during screening tests (20 animals). The blood from these cows was collected two months after they had been vaccinated against bovine viral diarrhea. These cows had reproductive problems, such as the inability to become pregnant after several attempts of artificial insemination or abortion. An ultrasound examination at 35 days of gestation showed the presence of a fetus, but at 90 days it was no longer present. Group 2 (control) comprised healthy cows that had tested negative for BVD and had no reproductive problems (20 cows).

### 2.2. Cell Culture

The peripheral blood lymphocytes were cultured in vitro in a Lymphogrow growth medium for 72 h at 38.5 °C (5% CO_2_, with stable humidity). At 69 h of the culture, colchicine was added (2.5 µg mL^−1^). At 24 h, BrdU (5-bromodeoxyuridine) was added to the cultures for the SCE assays (10 µg mL^−1^), and at 65 h, BrdU was added to the cultures for the FS test (5 µg mL^−1^). Potassium chloride (0.65% KCl) was used as a hypotonic solution. The cells were fixed using Carnoy’s fixative (3:1 methanol-acetic acid). The suspension was spotted on microscope slides and then used for the detection of damage by the SCE and FS assays.

### 2.3. Sister Chromatid Exchange Assay

The samples prepared for the detection of the sister chromatid exchanges were digested with 0.01% RNase for 1 h and then incubated in a solution of 0.5 × SSC with a Hoechst solution for 1 h. The samples were subjected to irradiation for 30 min, incubation overnight at 4 °C, UV irradiation for 30 min, incubation at 58 °C, and Giemsa staining for 1 h. The stained sister chromatid exchanges were counted in 20 metaphases from each individual.

### 2.4. Fragile Sites Assay

The microscope slides were incubated in a Hoechst solution with 2 × SSC (1 µg mL^−1^) and then subjected to UV irradiation for 1 h, incubation in 2 × SSC at 65 °C for 1 h, and Giemsa staining for 1 h. Chromatid breaks, chromatid gaps and chromosome breaks were identified. Twenty metaphases from each individual were examined.

### 2.5. Comet Assay (Single Cell Gel Electrophoresis)

DNA damage in the lymphocytes was identified using single cell gel electrophoresis. The lymphocytes were isolated using Histopaque-10771 (Sigma-Aldrich Co. LLC; Irvine, UK). The slides coated with a layer of 0.5% NMP (normal melting point) agarose gel were spotted with the lymphocytes mixed with a 0.5% LMP (low melting point) agarose gel and then embedded in LMP agarose. The samples prepared in this manner were subjected to alkaline lysis overnight (2.5 M NaCl, 100 mM Na2EDTA, 0.4 M Tris-HCl, 1% sodium N-lauroylsarcosinate, 10% Triton X-100, 1% DMSO, pH = 10) to release the DNA from the cell and remove the proteins. This was followed by alkaline denaturation in an electrophoresis solution for 30 min, after which the lymphocytes were subjected to electrophoresis (25 V, 300 mA, 20 min). Following electrophoresis, the lymphocytes were neutralized using Tris-HCl and stained with ethidium bromide. The DNA integrity was determined on the basis of the percentage content of the DNA in the tail of the comet (%T DNA). Fifty cells were analyzed for each animal.

### 2.6. Analysis

An Olympus BX50 microscope was used for microscopic analysis. The MultiScan image analysis software (v. 8.08) from Computer Scanning Systems was used to analyze chromosome damage, identified in the form of sister chromatid exchanges and fragile sites. The CASP 1.2.2 software was used to analyze degraded DNA in the lymphocytes identified by the comet assay. The changes observed the in cells were classified according to Gedik’s scale: N—no DNA damage or less than 5% damage in the comet tail, L—low level of damage (5–25%), M—intermediate damage (25–40%), H—high level of damage (40–95%), and T—over 95% DNA damage [60].

The results were subjected to statistical analysis using the Statistica 12.5 MR1 PL software. The influence of the group and individual on the incidence of chromosomal instabilities (SCE, FS, and SCGE) was analyzed using a one-way analysis of variance. Significant differences between the means for a given type of instability within the factors were assessed using Tukey’s test (*p* < 0.05). A student’s *t*-test was used to compare the means between groups 1 and 2.

## 3. Results

**Figure 1:**
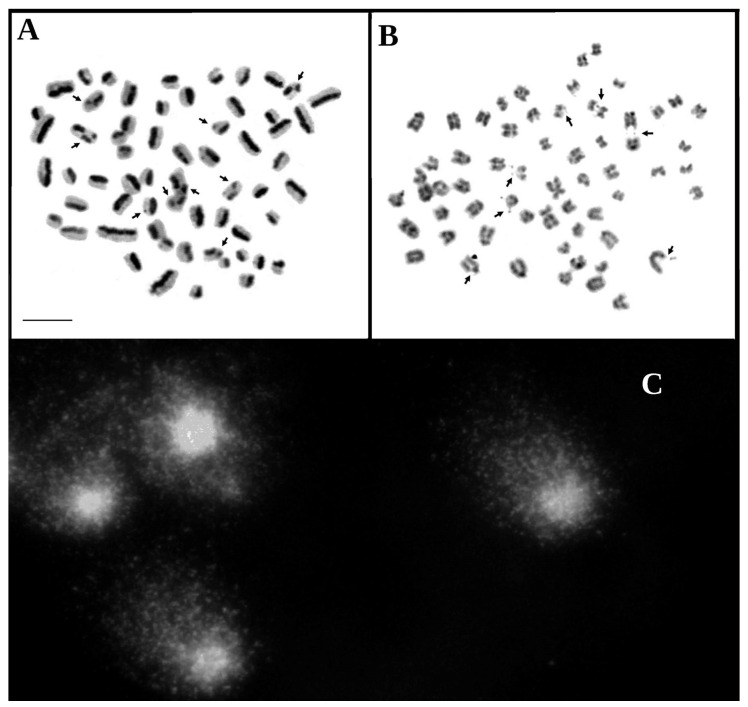
Mitotic chromosomes from the cows in group 1 in the metaphase stained by the SCE assay (**A**) and FS assay (**B**), and the cell nuclei of the lymphocytes subjected to the comet assay (**C**). The damage is marked with arrows. Scale bar 10 µm.

**Figure 2:**
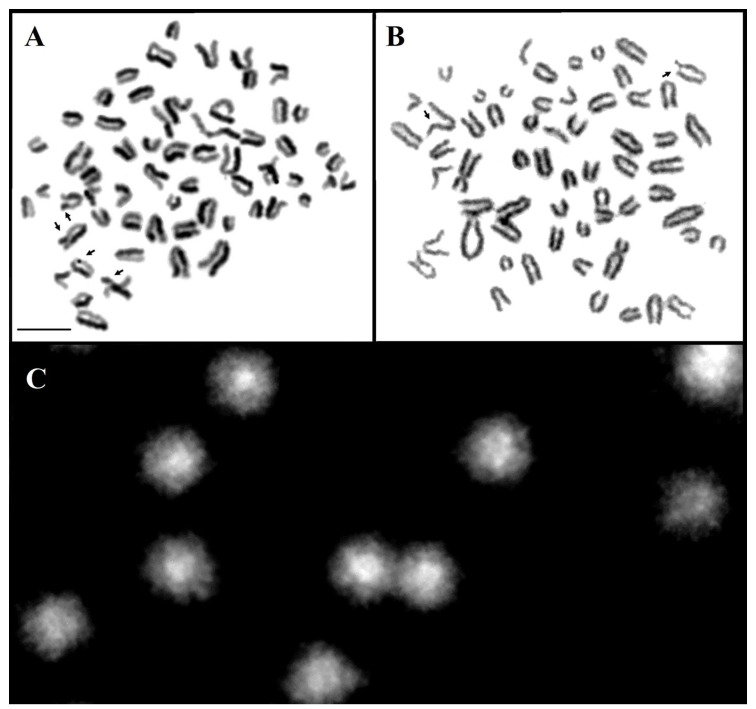
Mitotic chromosomes from the cows in group 2 in the metaphase stained by the SCE assay (**A**) and FS assay (**B**), and the cell nuclei of the lymphocytes subjected to the comet assay (**C**). The damage is marked with arrows. Scale bar 10 µm.

In the cows selected for cytogenetic testing, damage to the genetic material was identified using the SCE, FS, and SCGE assays. Figure 1 presents images of the metaphase chromosomes and nuclei of the lymphocytes subjected to the three assays in the cows with reproductive problems. Figure 2 presents images of the metaphase chromosomes and nuclei of the lymphocytes subjected to the three assays in healthy cows.

The average rate of SCE/cell was 6.7 ± 2.3. It was 8.6 ± 1.2 for G1 and 4.7 ± 1.2 for G2. Statistically significant differences were noted between group 1 and group 2 (*p* ≤ 0.00). Differences in the average frequency of SCEs were observed between the cows within each group, but they were statistically significant only between certain individuals (Table 1). The highest frequency of SCEs was noted in individual no. 6, and the lowest in no. 10 (group 1). In the second group, the highest frequency of SCEs was noted in individual no. 36, and the lowest in no. 27. The average rate of FS/cell was 5.0 ± 2.3 (G1 6.9 ± 1.4; G2 3.1 ± 1.0), and these differences were statistically significant (*p* ≤ 0.00). In this assay, differences in the frequency of damage were observed between the animals within each group, but they were only statistically significant between certain cows (Table 1). The most FSs were found in cow no. 6, and the fewest in no. 10 (group 1). In the control group, the highest frequency of FSs was noted in individual no. 36, and the lowest in no. 27. The average %T DNA in the cows was 13.1 ± 11.7; it was 22.7 ± 6.0 for G1 and 3.5 ± 7.4 for G2. The differences observed in the amount of fragmented DNA between group 1 and group 2 were statistically significant (*p* ≤ 0.00). Similar to the previous assays, the varying frequency of damage was identified by the comet assay. The differences were only statistically significant between certain cows (Table 1). The most damage was observed in individual no. 6, and the least, similar to the previous assays, in no. 10 (group 1). In group 2, the highest level of damage was noted in individual no. 29, and the lowest in no. 37 (Table 1). Based on the additional criterion applied, i.e., Gedik’s scale, the animals were classified as N (16 cows), showing no DNA damage or less than 5% damage in the comet tail; L (22 cows), with a low level of damage (5–25%); and M (two cows), with intermediate damage (25–40%). In G1, 18 cows were classified as L and two cows as M, while no animals were classified as N; H, i.e., with a high level of damage (40–95%); or T, with over 95% DNA damage. Two levels of damage were noted in the healthy cows (G2): N in 16 cows and L in four cows. Both of these levels were associated with a low level of DNA damage.

## 4. Discussion

The International Committee on Taxonomy of Viruses has identified 11 species of pestiviruses, designated A through K, and additionally divided into subgroups (subgenotypes) [8,61,62]. One of these is the BVD virus, which adversely affects animal health and reproduction. Bovine viral diarrhea is regarded as a disease with a moderate to high risk of spreading around the world [63,64]. One of the preferred methods for combating this disease is vaccination, which is relatively inexpensive and effective. The most important effect of BVD vaccination is the protection of heifers and cows against transplacental infection, which results in persistently infected animals. Calves are also vaccinated to protect them against clinical infection and minimize the risk of infection [8]. Systematic vaccination prevents new infections, reduces shedding of the virus, and produces herd immunity [30,65,66]. According to Sozzi et al. [8], however, vaccination does not provide sufficient protection against BVD, which would explain why the disease spreads so rapidly despite vaccination. Zimmer et al. [67] and Grooms et al. [68] also claimed that vaccination with a predefined strain of BVD may not provide adequate protection against other strains of the virus. Many European countries have introduced compulsory programs for the control of every animal in the cattle population in order to eradicate the virus [9,28,69,70,71,72]. Unfortunately, the negative effects of the virus include problems with fertility, damage to the developing fetus, and the birth of weak calves or calves with congenital defects. They can also lead to the death of the fetus or to mummification and abortion, which prolongs the calving interval and is often the cause of culling. These consequences entail economic losses for dairy cattle farmers. Due to their low immunity, the infected animals are vulnerable to other diseases as well. For this reason, it is important that the animals should have a high level of genome integrity and stability. This enables rapid defense against infection and correct cellular responses to DNA damage arising during replication and transcription. Maintaining the animals’ health is crucial for dairy cattle farmers [6]. 

Fertility problems are one of the most common causes of culling in dairy cattle herds. The most common reproductive disorders include a prolonged calving-to-first-estrus interval and calving-to-first-service interval, less pronounced external signs of estrus, ovulation and luteolysis disorders during the estrous cycles, reduced conception rates, abortions, prolonged estrous cycles, deterioration of the oocyte quality, and dystocia [19,73,74,75]. Cows have been bred for milk yield over many years at the expense of reproductive traits. A negative correlation between reproductive traits and milk production traits has been reported by numerous researchers, such as LeBlanc [76], Pimentel et al. [77], Zink et al. [78], Yamazaki et al. [79], and Weigel et al. [80]. In recent years, many breeders have emphasized not only high production, but functional and reproductive traits as well [81,82,83]. 

Genetic tests, especially cytogenetic assays, are helpful tools for assessing genomic stability and integrity. Many chromosome instabilities reduce fertility in cows [84,85,86,87,88,89], and structural and numerical mutations are among the instabilities which cause reproductive problems [89,90,91,92]. Scientists are increasingly assessing the genomic stability using cytogenetic assays, which identify chromosome instability arising as a result of malfunctioning cellular mechanisms, such as replication, transcription, damage repair, and checkpoints [93,94,95]. These errors also adversely affect reproduction and can lead to economic losses [96]. Cytogenetic assays are highly sensitive and useful tools for detecting damage to genetic material [97]. The assays used in our study provided information on the level of susceptibility of the DNA and chromosomes to harmful exogenous and endogenous factors and on the functionality of replication, transcription, and control and repair mechanisms. These biomarkers can be used successfully to prevent disease during its course and assess animals’ genetic resistance [98]. 

Unfortunately, there are no reports on the identification of genomic instability in animals infected with BVD. Many researchers have linked the presence of this virus to reproductive problems, such as disruptions in the development of egg cells, infertility, reduced conception rates, reduced rates of successful artificial insemination, or abortion [99,100,101,102]. These researchers noted a loss of pregnancies in the early stage of gestation (10 to 90 days). Similar observations have been reported by Oguejiofor et al. [103] and Subekti et al. [104], who found that the BVD virus kills egg cells, embryos, and fetuses. It is highly likely that during meiosis in egg cells, as well as in the cell cycle of fetal somatic cells, excessive damage of various types is generated in genetic material, which is a teratogenic effect of the virus. The most severe complications in the systems and organs are observed in the first days of pregnancy and result in early abortion. Many viruses use two strategies for infecting the host, known as ‘hit and run’ and ‘hit and stay’. The BVD virus behaves this way as well. This results in the impairment of innate and acquired immunity [72]. The lethal effects of the virus manifest when the immune system is impaired. When the virus penetrates the cells in a state of immunosuppression, it causes the formation of new virions, which attack further cells, thereby impairing DNA synthesis and dysregulating the cell mechanisms that ensure normal cell division, such as checkpoints and DNA repair mechanisms. This results in accumulated damage which becomes established in the M phase. Immune deficiencies are linked to disorders in the repair of DNA strand breaks, leading to disturbances in the integrity and stability [105]. According to Nehra et al. [106], the rapid spread of viral diseases in cattle should be addressed by implementing various methods, technologies, and procedures to prevent the expansion of viruses and minimize negative health consequences. Hence, our study is a valuable contribution to the dissemination of information on the detection of latent genomic instability in cows with BVD. Abnormalities in the course of cell division and the absence of effective mechanisms minimizing errors result in various anomalies in animals. 

An increased frequency of chromosome instability has been observed in Bovidae with hemimelia, amelia, or polymelia [107,108,109,110,111,112], in goats with hemimelia [113], and in chickens with chondrodystrophy [114]. According to Sonoda et al. [115], Wójcik et al. [98], and Dezfouli [116], the level of damage generated should not exceed 10 lesions per cell. According to Wilson and Thompson [45], the frequency of damage in healthy animals should be no more than three lesions per cell, and deviations from this standard are indicative of pathological changes in the body [98]. These numbers are species-dependent, as SCEs and FSs are conserved traits in the species. The observed frequency in the present study in the cows with reproductive problems was 7.7 to 9.2 SCE/cell, with a predominant frequency above 8.5, and 5.7 to 8.6 FSs, with a predominant frequency above 6.5 (average: SCE 8.6, FS 6.9). These frequencies were twice as high as in the control group (average: SCE 4.7, FS 3.1). Wójcik and Szostek [117] reported a frequency of 6.4 SCEs and 4.2 FSs per cell in healthy HF cows. These frequencies were lower than those in the present study in the cows from group 1, which may indicate that the BVD virus may have increased the frequency of this type of damage. In the control group, consisting of healthy cows, we observed a low frequency of instability in comparison to group 1. 

Various researchers have obtained varying results, ranging from two to eight FSs/cell [116,118,119,120,121,122,123,124]. These values may have been influenced by a variety of factors, such as the animals’ breed or living environment. Similarly, the frequency of FSs in cattle reported by different authors varied from 0.2 to 4.5/cell [97,107,125,126,127,128]. Low instability rates were observed by Wójcik et al. [129] in pregnant recipient cows taking part in OPU/IVP in vitro fertilization: 5.0 SCEs, 3.2 FSs, and 3.8% T DNA. That study was one of the few reports of the use of the SCGE assay to identify spontaneous genomic instability in HF cows. In the present study, the mean level of %T DNA was 22.7 in the cows from group 1, which was significantly higher than the level in the control group (3.5) and the results presented by Wójcik et al. [129], which may indicate that the BVD virus negatively affected the DNA integrity. Other researchers have used the SCGE assay to assess the degree of genotoxicity and mutagenicity of the physical and chemical factors and observed a high frequency of DNA damage [39,130,131,132,133,134,135]. This assay has also been used to assess the degree of DNA fragmentation resulting from the clastogenic effect of the papilloma virus *Bovine papillomavirus* [136], and it revealed an increased level of damage to genetic material. In an investigation of apoptosis in the lymphocytes and neutrophilsin cattle with lymphosarcoma, this assay revealed a decrease in apoptotic cells in sick animals compared to healthy ones, which may indicate the “immortality” of cancer cells [137]. The SCGE assay has also been used to estimate the degree of DNA fragmentation in the lymphocytes of cows affected with acute mastitis [138]. The researchers reported an accelerated rate of DNA damage in sick animals in comparison with healthy cows. Thus, according to many researchers, the comet assay is a very useful genetic tool for assessing DNA integrity. In the present study, it proved to be the most sensitive biomarker, providing highly reliable results and indicating the state of the animal’s health, which was influenced by the level of DNA damage.

The lack of any information pertaining to the cytogenetic assessment of the genome of cows infected with the BVD virus and its pathogenic effect on reproduction prompted us to undertake this research. The present study is a pilot study. It would be very interesting to assess seronegative and seropositive animals with and without reproductive problems. The results would enable more in-depth research into the pathogenicity of the BVD virus, which is responsible for cosmopolitan diseases in cattle, with subclinical and clinical symptoms, and has a significant impact on animal production and the economy. Although it will be time consuming, the research will be continued in the future in order to provide more complete information on the negative impact of BVD on reproduction in cows.

## 5. Conclusions

The identified genomic instability may be indicative of an animal’s health status and its response to the BVD virus as a mutagenic factor, as we observed a lower frequency of gene instability in healthy individuals. There is a need for further research enabling a thorough assessment of the pathogenic effects of the BVD virus on the genomes of animals. The cytogenetic assays used to assess the stability and integrity of the cows’ genetic material showed great potential, especially the comet assay. Unfortunately, the changes in the chromosomes not only caused changes in the gene expression, but also affected the topology and dynamics of the genome. This is crucial for maintaining the animal’s health and reproductive capacity, which are the factors that determine the productivity of cows, as their resistance largely depends on a stabile genome. The assessment of genomic stability and integrity would significantly supplement and broaden genome selection protocols and could become one of the tools for practical selection. Given the negative impact of chromosomal abnormalities on the reproductive capacity of animals, cytogenetic assays could be used as a diagnostic tool in breeding and reproduction.

## Figures and Tables

**Table 1:** Number of instabilities identified (SCE, FS, and SCGE) in the cows. The means designated with different letters are significantly different at *p* < 0.05.

	Group 1					Group 2			
Cow	SCE	FS	SCGE		Cow	SCE	FS	SCGE	
	Mean ± SD			Gedik’s Scale	Mean ± SD				Gedik’s Scale
1	8.2 ^abc^ ± 0.8	6.1 ^a^ ± 1.2	19.8 ^ab^ ± 5.0	L	21	4.3 ^abc^ ± 1.0	2.7 ^ab^ ± 0.8	2.8 ^abc^ ± 5.3	N
2	9.0 ^abc^ ± 1.0	8.0 ^de^ ± 1.1	21.8 ^abc^ ± 5.7	L	22	5.0 ^abcd^ ± 1.1	3.4 ^bc^ ± 0.9	4.7 ^abc^ ± 10.1	N
3	9.2 ^bc^ ± 1.5	8.4 ^e^ ± 1.0	26.4 ^ef^ ± 5.1	L	23	5.3 ^bcd^ ± 1.3	3.4 ^bc^ ± 0.8	6.2 ^c^ ± 9.9	L
4	8.8 ^abc^ ± 1.6	7.5 ^bcde^ ± 1.5	21.3 ^ab^ ± 4.5	L	24	5.1 ^abcd^ ± 1.1	3.2 ^abc^ ± 0.7	4.1 ^abc^ ± 9.4	N
5	9.0 ^bc^ ± 1.2	8.2 ^e^ ± 1.4	28.0 ^fg^ ± 5.7	M	25	4.8 ^abcd^ ± 1.0	3.3 ^bc^ ± 0.9	3.3 ^abc^ ± 7.6	N
6	9.5 ^c^ ± 1.4	8.6 ^e^ ± 1.0	30.3 ^g^ ± 4.8	M	26	3.9 ^a^ ± 1.1	2.5 ^ab^ ± 0.8	1.1 ^a^ ± 3.2	N
7	8.3 ^abc^ ± 1.1	6.4 ^ab^ ± 1.0	20.0 ^ab^ ± 7.4	L	27	3.9 ^a^ ± 1.0	2.3 ^a^ ± 0.8	1.0 ^a^ ± 3.3	N
8	8.5 ^abc^ ± 0.8	6.6 ^abc^ ± 1.1	20.8 ^ab^ ± 5.4	L	28	5.0 ^abcd^ ± 1.2	3.4 ^bc^ ± 0.8	4.1 ^abc^ ± 7.6	N
9	8.6 ^abc^ ± 1.1	6.5 ^abc^ ± 1.3	22.6 ^bc^ ± 4.6	L	29	5.3 ^bcd^ ± 1.1	3.2 ^abc^ ± 0.9	6.3 ^c^ ± 8.2	L
10	7.7 ^a^ ± 1.4	5.7 ^a^ ± 1.3	18.6 ^a^ ± 4.9	L	30	4.8 ^abcd^ ± 1.3	2.9 ^ab^ ± 0.6	4.0 ^abc^ ± 8.5	N
11	7.9 ^ab^ ± 1.3	6.3 ^ab^ ± 1.6	19.9 ^ab^ ± 5.9	L	31	5.6 ^cd^ ± 1.2	3.9 ^cd^ ± 1.2	5.9 ^c^ ± 7.8	L
12	8.3 ^abc^ ± 1.7	6.5 ^abc^ ± 0.8	20.8 ^ab^ ± 4.4	L	32	4.5 ^abcd^ ± 1.0	2.7 ^ab^ ± 0.9	3.0 ^abc^ ± 6.1	N
13	8.0 ^ab^ ± 1.4	5.9 ^a^ ± 1.3	20.7 ^ab^ ± 4.4	L	33	4.7 ^abcd^ ± 1.0	2.9 ^ab^ ± 0.8	3.7 ^abc^ ± 7.8	N
14	8.8 ^abc^ ± 0.9	6.8 ^abcd^ ± 1.0	24.5 ^cde^ ± 4.9	L	34	4.3 ^abc^ ± 1.0	2.7 ^ab^ ± 1.1	2.0 ^ab^ ± 5.1	N
15	8.6 ^abc^ ± 1.0	6.6 ^abc^ ± 1.0	22.4 ^bc^ ± 4.0	L	35	4.5 ^abcd^ ± 1.2	2.9 ^ab^ ± 0.7	2.6 ^abc^ ± 6.8	N
16	8.4 ^abc^ ± 4.0	6.3 ^ab^ ± 1.5	22.3 ^bc^ ± 4.9	L	36	5.7 ^d^ ± 0.9	4.7 ^d^ ± 0.7	6.9 ^c^ ± 9.8	L
17	8.8 ^abc^ ± 1.4	6.7 ^abcd^ ± 1.0	22.8 ^bcd^ ± 4.9	L	37	4.1 ^ab^ ± 1.2	2.7 ^ab^ ± 0.8	0.9 ^a^ ± 3.2	N
18	8.9 ^abc^ ± 1.0	7.8 ^cde^ ± 1.0	24.4 ^cde^ ± 5.9	L	38	4.6 ^abcd^ ± 1.3	3.0 ^ab^ ± 0.9	3.3 ^abc^ ± 6.6	N
19	8.7 ^abc^ ± 1.1	6.8 ^abcd^ ± 1.3	21.6 ^abc^ ± 4.6	L	39	4.5 ^abcd^ ± 1.2	3.1 ^abc^ ± 0.9	1.7 ^ab^ ± 3.8	N
20	9.1 ^bc^ ± 1.1	7.5 ^bcde^ ± 1.2	26.2 ^def^ ± 6.5	M	40	4.8 ^abcd^ ± 1.0	3.0 ^abc^ ± 0.8	2.9 ^abc^ ± 7.9	N

## Data Availability

Data is contained within the article.
